# Lasting Deficiencies in Vergence Eye Movements in Patients with Peripheral or Central Vertigo: Improvements After Four Sessions of REMOBI Neurotraining and Associated Functional Benefits

**DOI:** 10.3390/brainsci14111131

**Published:** 2024-11-08

**Authors:** Zoï Kapoula, Ganesan Aakash, Guèrin Rèmi, Alain Bauwens, Benoit Martiat, Valentine Leonard

**Affiliations:** 1 LIPADE, University Paris Cite, 75006 Paris, France; 2 Eye Analytics & Rehabilitation Research Group, 75015 Paris, France; aakuganesan@gmail.com; 3 Unité de Réhabilitation Fonctionnelle des Vertiges, Hôpital Saint Thérèse (VIVALIA), 6600 Bastogne, Belgium; guerin.orthoptie@gmail.com (G.R.); alain.bauwens@chuuclnamur.uclouvain.be (A.B.); info@orlbastognearlon.be (B.M.); 4 CHU UCL Namur ENT Service, 5000 Namur, Belgium; valentine.leonard@chuuclnamur.uclouvain.be

**Keywords:** vertigo, vergence eye movements, vergence rehabilitation, posture

## Abstract

The vestibular function is in synergism with the oculomotor vergence. Vertigo may be related to vergence disorders and conversely, vestibular pathologies may affect vergence. To consolidate this hypothesis, we conducted a study at the vestibular orthoptic clinic of the Bastogne Hospital. Fourteen patients with vertigo history appearing 2 weeks to 8 years ago, aged 30 to 65 years were studied; at the moment of the eye movement study, no patient had acute attack of vertigo. The origin of vertigo varied (Meniere’s disease, organic pathology, sensitivity to visual movement). An assessment with objective measurement of vergence (single-step protocol) was carried out with the REMOBI technology coupled with binocular video-oculography in sitting and standing positions. Four neuro-rehabilitation sessions of vergence eye movements were performed with the double-step in-depth protocol, alternating sitting and standing positions to involve different postural and vestibular functions. An assessment of vergence was done again 1 to 2 months later. The initial assessment revealed problems of magnitude and/or speed or variability of vergence for 11 of the patients relative to controls (published by the group in previous studies). After neuro-rehabilitation, an improvement was observed in eight patients. Patients reported a clear improvement of their self confidence in moving in the space. Posture measures done before rehabilitation comparing eyes fixating or closed or while making near–far vergence eye movements indicated lower medio-lateral acceleration when doing vergence eye movements in patients with vertigo history of functional origin. The results are in favor of the hypothesis of a symbiosis between vergence and vestibular function and the interest of diagnosis and rehabilitation of the vergence disorder in patients with vertigo history in the absence of acute vertigo attack.

## 1. Introduction

The eye movement in vergence makes it possible to adjust the angle of the visual axes according to the depth with which the object of interest is located. The eyes move in opposite directions: to increase the angle if the object is close (convergence) or to decrease it if the object is far away (divergence). Multiple stimuli participate in the triggering and execution of movements, such as binocular disparity, blurring and resulting accommodation, sensation of proximity, and monocular depth indices such as visual perspective [1,2]. The control of these complex eye movements involves the occipital, parietal, and frontal cortex [3,4,5,6], the superior colliculus [7,8], the brainstem [9], and the cerebellum [10,11,12]. The cerebellum is involved in the control of vergence, both in real time (direct effect) [13,14,15] and delayed (adaptation effect) [16,17]. A brain function imaging study has provided evidence for the existence of specific neuronal circuits involved in vergence and saccades, namely, in the frontal visual fields and the brainstem [18]. In the brainstem, convergence and divergence are generated by specific phasic, tonic, and tonic-phasic cells situated in the mesencephalic reticular formation [6]. Thus, the divergence is not simply a passive relaxation of convergence, but a distinct neurophysiological process.

Vergence eye movements are essential for binocular fusion, depth perception, and stereopsis. Moreover, the gain of the vestibular response, which makes it possible to ensure the stability of the image on the retina during a head movement, in particular that of the translational vestibulo-ocular reflex (tVOR), depends on the distance of the visualization [19]; the gain increases in near vision and decreases in far vision. Adjustment of the gain could be modulated by vergence signals. Migliaccio et al. (2004) [20] showed that in humans the modulation of the horizontal rotational vestibulo-ocular reflex (VOR) by vergence disappears in the case of partial destruction of the inner ear by gentamicin. The authors hypothesize that irregular afferents from hair cells in the central zone of the ampullary crest provide the necessary signal used centrally to increase the VOR gain with vergence. However, at the central level, the VOR is very quickly triggered; it has been suggested that the involvement of the vergence could act via the cerebellum, which learns to provide a vergence pulse combined with the VOR (see model proposed by Coenen) [21]. In connection with such conceptual models of vergence–VOR interaction, clinical studies have shown that patients with supranuclear palsy (PSP) have both vergence disorders and non-increase in tVOR gain with near vision [22,23,24]. Ramat and Zee [25] studied the dynamics of oculomotor responses to sudden head translation movements in healthy patients and reported the adjustment of tVOR to vergence angle; they also observed that jerks were an integral part of the adjusted tVOR with viewing distance.

Studies of children and adolescents with vestibular symptoms (vertigo, headaches), for which no vestibular signs had been clinically observed, measured abnormalities of saccades, and especially vergence eye movements [26,27,28,29,30]. In another study of adult patients with bilateral idiopathic vestibular loss, vergence eye movements were measured to be particularly deficient relative to control adults; yet, postural control in quiet stance was found to be better when the patients were asked to make actively near–far vergence eye movements than when fixating either at near or at far [31]. Thus, in addition to the link between vestibular function and vergence, this latter study indicates an active positive action of vergence eye movements on postural control. Reciprocally, Erkelens et al. [31] had already shown that vergence movements were faster and better directed to a target when the person was in action (e.g., standing) than when they remained motionless (seated). Thus, evidence exists for mutual interactions between vergence and vestibular function and vertigo symptoms, as well as on posture and vergence eye movements.

Taking into account the concept of reciprocity in the symbiotic interaction between vergence, vestibular function, and postural control, the present study analyzed the properties of convergence and divergence movements in adult patients with a vertigo history caused by confirmed vestibular involvement; vergence eye movements were studied in both seated and standing positions. The rationale behind this was the following: In the seated position, the vestibular system is less active due to greater stability and fewer balance demands. In standing, the system is more engaged, coordinating with proprioception and the vestibulo spinal reflex to maintain balance as the body’s center of gravity is higher. Standing also increases postural sway, requiring more vestibular control. Moreover, an additional test of postural control in quiet stance was run to assess the impact of eye movements on postural control (the person was either fixating, or making active vergence movements [near–far], or with closed eyes). The ensemble of these tests aimed to further investigate the intricacies of vestibular, postural, and vergence interactions in adults with a history of vertigo of variable origin.

An additional important question addressed in this studywas the possibility to improve vergence eye movements in such patients via a rehabilitation paradigm, the double-step in-depth paradigm that was found to be very efficient in young adults with isolated vergence disorders [32]. Thus, we carried out the initial functional exploration of vergence eye movement in sitting or standing position, the posturography tests, then we offered them four vergence rehabilitation sessions and finally, we objectively reassessed the vergences in standing or seated positions of all these patients after the four reeducation sessions. Unfortunately, for technical reasons, posturographywas not possible after the vergence rehabilitation. All eye movement tests and rehabilitation were done with the REMOBI technology (see Section 2). To summarize, the main hypothesis is that due to vergence–vestibular–postural interactions, vergence deficiencies may be frequent in adult patients with a history of vertigo and could be dependent on posture, whether seated or standing. The second hypothesis is that vergence can be improved in such patients through vergence double-step protocol training.

## 2. Methods

Fourteen vertiginous patients participated in this study. The “vertigo” symptom was the essential criterion of inclusion. These patients were initially assessed on the Ear, Nose, and Throat hospital service plan and underwent a complete orthoptic examination. Vestibular function was assessed by videonystagmoscopy (VNS) in complete darkness. None of these patients were in acute attacks of vertigo. In other words, none of them had spontaneous nystagmus that could be observed with bare eyes. The ocular fixation index was therefore correct, that is to say that the visual fixation completely inhibited any spontaneous vestibular nystagmus observed under VNS. The protocol was approved by the ethics committee I.F.A.C. n° 152, and patients gave their written consent.

Table 1 lists four types of vertigo symptoms: Positional vertigo: vertigo triggered and felt during a supine position. Vestibular Asthenopia: all visual symptoms, relatively permanent related to the movement. Discreet vertigo: brief, episodic vertigo, perceived several times a day. Chronic vertigo: recurrent vertigo with a rotational nature and evolving by attacks.

The vestibular state gathers the observations made under videonystagmoscopy, the eye being filmed in the dark, under infrared camera. It indicates either the objective characteristics of an asymmetry of the vestibular balance or, in the absence of objective signs, a subjective notion of sensory discomfort related to movement. The presence of spontaneous nystagmus indicates an uncompensated vestibular state. Hyporeflectivity is the residual sign of vestibular hypofunction observable during the rotational pulse test protocol (6/14 patients).

Fusional amplitudes for convergence (C) and divergence (D) were measured by the orthoptist with the Berens horizontal prism bar, for far viewing distance (C and D) and for near view distance (C’ and D’). The NPC, near punctum of convergence, was also measured. Many of these orthoptic values were below reference values (see legend of Table 1).

### 2.1. Oculomotor Tests

#### 2.1.1. REMOBI, Vergence Test (Single-Step)

In order to objectively evaluate the presence of abnormalities of vergences, we used the device REMOBI by Orasis-Ear, Paris, France. REMOBI (PCT WO2011/073288) is a trapezoid-shaped horizontal device, equipped with visual-acoustic elements (diodes and buzzers) and embedded software. REMOBI is a device that contains specific and different algorithms, on the one hand for the assessment (diagnosis of anomalies of vergences), on the other hand, for their neuro-rehabilitation of vergence movements. The patient’s eye movements were recorded in this study with the EyeSeeCam video-oculography binocular with a frame rate of each eye at 220 Hz (see https://eyeseecam.com, Munich, Germany). The protocol to be described below is part of the patent (Functional differential exploration and rehabilitation of binocular motor coordination in individuals suffering from vertigo, CNRS, PCT, WO2022/074132, Europe 7 October 2021).

**Figure 1:**
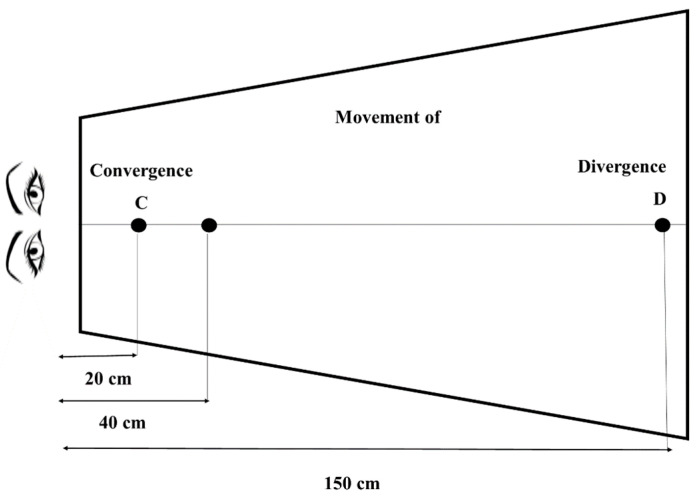
Vergence assessment carried out with REMOBI. In an entire 2.5 min test, patients randomly performed 40 movements: 20 convergence movements, and 20 divergence movements. F is the position of initial fixation of LED, C is the target position requiring a convergence movement, and D is the target location requiring divergence movement.

Single-step vergence test: the vergence movements were tested with the following paradigm: at each test, a fixation diode was presented in the median plane at 40 cm of the subject during 1500 ms, preceded by a short beep, which served as a warning attentional; then, a second diode appeared 1200 to 1800 ms after the beginning of the first presentation, located 1.50 m from the subject, or located 20 cm from the subject. In the first case, a divergence movement had to be madeto fixate further away; in the second case, a convergence movement had to be made to fixate a diode closer (see Figure 1). This vergence test consisted of 40 randomly interpolated trials containing 20 convergence and 20 divergence trials. The test was run twice in sitting or standing position.

#### 2.1.2. Vergence Double-StepRehabilitation Protocol

For the rehabilitation of vergence, we used another embedded algorithm ofREMOBI, the so-called vergence protocol in double-step, detailed in the study by Kapoula et al., 2016 [32]. During this protocol, the subject must make a vergence movement towards the first target LED. This target, however, is presented only for a very short time and is replaced by a second target, even closer to convergence training and even more distant for divergent training, forcing the subject to quickly reprogram the initial movement.

**Figure 2:**
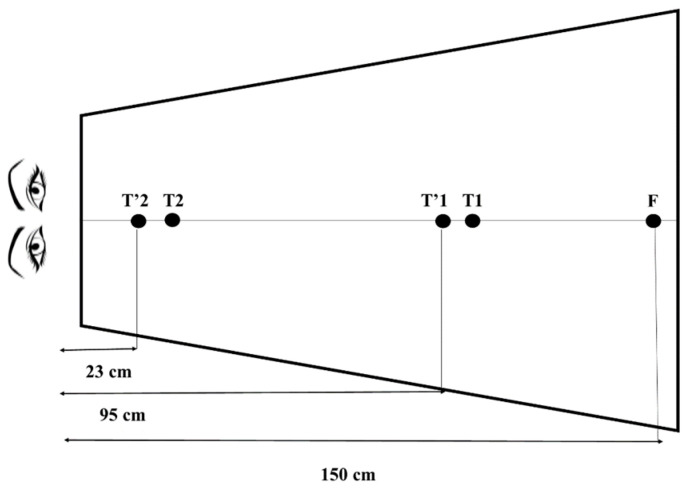
Double-step rehabilitation protocol for vergence movements: For convergence training, the initial fixation LED (F) appeared at 150 cm for 1600 ms. It was immediately followed by a target LED (T1 or T’1), after 200 ms this LED was switched off and the second final target LED (T2 or T’2) was presented for 1300 ms.

Typical convergence and divergence rehabilitation trials with the double-step paradigm are shown in Figure 2. Briefly, the target for convergence or divergence appeared first at a location (T1 or T’1); after 200 ms, presumably before the accomplishment of the vergence eye movement, the target stepped to a second location (T2 or T’2). Given that the vergence latency is between 160 and 250 ms, and vergence execution lasts between 350 and 550 ms [31]; it is almost certain that the second step of the target occurred before the initial vergence eye movement has been made. The results brought by Kapoula et al. (2016) [32] in students with vergence abnormalities demonstrated the efficiency of this method; indeed, after five sessions of 35 min, we confirmed an amplification and acceleration of convergences and divergences, lasting 18 months later.

The four rehabilitation sessions were performed with the REMOBI device by the service orthoptist. The sessions were held more or less weekly depending on patient availability. Eye movements were not recorded during these sessions, each lasting 20 min.

The rehabilitation of the vergences was done by alternating either the sitting position or the standing position. Each time, the REMOBI device was placed at eye level. In the standing position, the person leads the synergy between vergences and postural balance in a more active way, which results in a real reeducation combining the gaze and the vestibular and postural function. Each participant completed four rehabilitation sessions in 2 months.

The vergence single-step test (assessment) presented above, including oculomotor recording by video-oculography, was performed before the rehabilitation and 1 month after the end of the four rehabilitation sessions. The results are presented below.

### 2.2. Postural Testing

The body sway was measured with the small DynaPortMiniMod^®^ (McRoberts B.V. The Hague, The Netherlands) device (74 g) equipped with three intransverse, sagittal, and coronal orthogonally mounted accelerometers (AXXL202, Analogue Devices, Norwood, MA, USA), placed on a belt at the lumbosacral level and near the center of mass of the body. The frequency of sampling was set at 100 Hz. We measured the following parameters: normalized area (in mm^2^/s), mean power frequency (MPF), the root-mean-square of the medio-lateral body sway (RMS of M/L in mm), the root-mean-square of the antero-posterior body sway (RMS of A/P in mm),the RMS of M/L velocity (in mm/s), and the RMS of A/Pvelocity.

The posture of the patients was measured in quiet stance and under three conditions: while fixating at far (150 cm), while making vergence eye movements as described above in the vergence test, or with the eyes closed. Postural testing was done once, at the beginning of the study, because of technical problems preventing us from recording post-rehabilitation.

Due to the limited number of patients, statistical analysis of the eye movement data was done with non parametric Wilcoxon test comparing group means before and after rehabilitation, or between sitting and standing conditions. For postural data, similar testing was done, as well as Spearman correlation between postural and eye movement parameters.

## 3. Results

**Figure 3:**
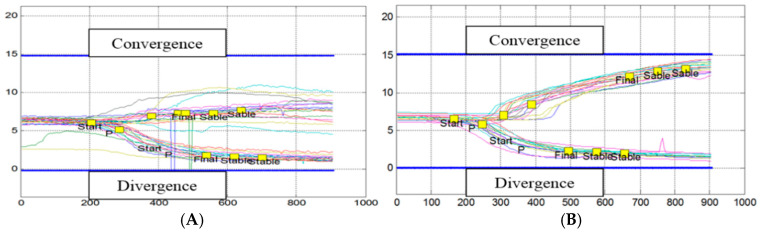
(**A**): Trajectories over time (in ms) of convergences and divergences from patient F55 in the standing position; the target appeared at zero time. After a latency of about 200 ms, the vergence movement begins. The trajectories are slow, variable, and hypsometric, especially for the convergence, which does not reach its target (indicated by the blue line). (**B**): Trajectories of the vergences of the patient F55 a month after the end of the rehabilitation with REMOBI. Convergence trajectories increase to almost reach the target (high blue line) and movement dynamics accelerate.

Figure 3A,B shows the trajectories of the vergences of the patient F55. Clinically, this patient complained of sensitivity to optic flow generating nausea, which appeared a few weeks before the first consultation, with impressions of fleeting instability, and of feeling a permanent flutter and discomfort in rapid movements of the head and eyes.

Figure 3A below shows the trajectories of its convergences (traced upwards) and the trajectories of its divergences (traced downwards) during the 2.5 min of testing in the standing position. The blue lines indicate the position of the respective target that the eyes should reach. This figure shows a strong hypometria, especially for convergence.

Figure 3B shows the trajectories of the patient a month after the four sessions of the rehabilitation. There is a significant acceleration and amplification of the convergence, and the movement was initially the most deficient. For divergence, there is a reduction in latency. Subjectively, the patient reported greater ease of visual exploration of her environment, a greater sense of stability, and increased confidence when walking.

**Figure 4:**
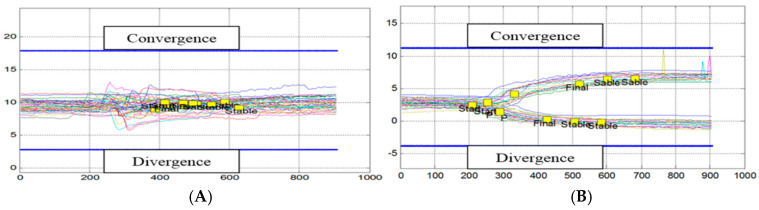
(**A**): Trajectories up for convergence targets, down for divergence targets. The trajectory is deficient in both cases (other notations as in Figure 3). (**B**): Improved trajectories after rehabilitation.

Figure 4A presents the results of patient number M61, suffering from an old and well documented left Meniere’s disease. He had undergone classical vestibular rehabilitation (consisting of nine rotary chair sessions). This rehabilitation was beneficial and avoided surgery with its labyrinthine risk. However, episodically, small attacks of low intensity and well tolerated by the patient reappeared; indeed, the periodically reproduced vestibular examination showed a state of marked left deficit with right directional gravity.

Interestingly, we see in Figure 4A a paucity of vergence. The patient tries to converge by performing earlier jerky movements, of low amplitude, reaching neither the target of the convergence (blue line up) nor the target of the divergence (blue line down). We then conclude in the existence of a major deficit of all vergence for this patient suffering from several years of Meniere’s disease that dramatically improves after rehabilitation.

### 3.1. Quantitative Analysis

#### EyeMovement

Eye movement parameters for convergence and divergence in the standing or seated position are shown in Table 1. Values in bold are abnormal: we used normative values as reference to those reported in another study ([32], Kapoula et al., 2016). We used the same reference values for seated and standing, as in this stage there is no reference on possible differences between seated and standing in normal conditions. Patient IDs are in bold for those patients who have a pathology functional origin of the vertigo, and the remaining patients had an organic origin (see legend of Table 1).

### 3.2. Eye Movement Results

**Figure 5:**
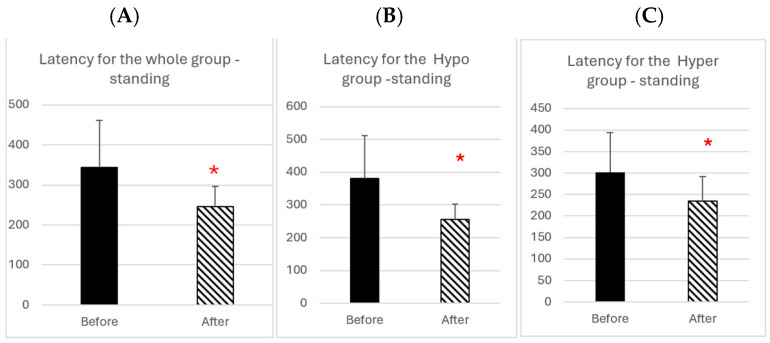
(**A**): Group mean latency of divergence in the standing position before and after rehabilitation. Vertical bars indicate standard deviation of the group means; * indicates statistical significant difference. (**B**): Divergence latency from the hypo group; (**C**): data from hyper group.

Figure 5 shows group mean values of latency of divergence in the standing condition before and after rehabilitation.

In Figure 5A, all patients are grouped together, while Figure 5B shows data from the subgroup of patients having an organic origin of vertigo, and Figure 5C shows patients having a functional origin of vertigo (the patients indicated by bold values in Table 2 and Table 3). Following rehabilitation, there is a statistically significant decrease in the latency for both the whole group and the two subgroups (Wilcoxon test W = 88, Z = 2.54, *p* = 0.0108 for the whole group, W = 27, Z = 2.2013, *p* = 0.027 for hypo group, W = 20, Z = 1.99, *p* = 0.046 for hyper group).

**Figure 6:**
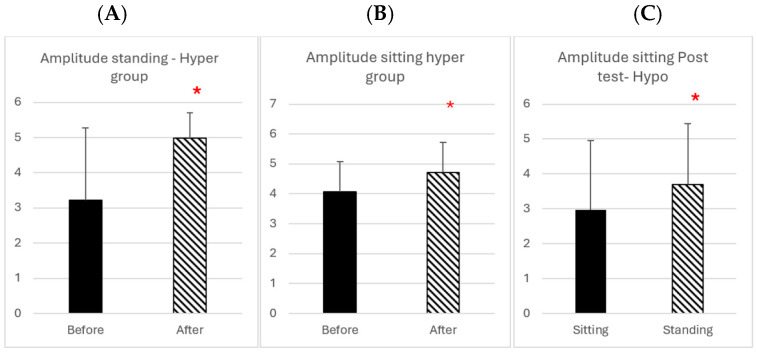
(**A**): Group mean amplitude of divergence before and after rehabilitation in the standing condition. (**B**): The amplitude of divergence in the before and after rehabilitation in the sitting condition for the hyper group whose vertigo was functional in origin. (**C**): Divergence amplitude after rehabilitation in the sitting vs. standing condition for the hypo group whose vertigo was organic in origin: asterisk indicate statistically significant differences.

Concerning the amplitude results, statistically significant differences were found for divergence only. Figure 6A,B shows the mean amplitude of the divergence before and after rehabilitation in the standing and seated condition, respectively. In Figure 6A, data from the hyper group show a significant increase of amplitude for divergence in the standing condition (Wilcoxon W = 82, Z = 2.201, *p* = 0.027); a similar increase of amplitude of the divergence after rehabilitation is also found in the seated condition in Figure 6B (Wilcoxon shows W = 26, Z = 1.99, *p* = 0.046).

In contrast for the patients with an organic origin of vertigo (hypo), following rehabilitation, the amplitude of divergence was higher in the standing than the seated condition in Figure 6C (Wilcoxon shows W = 20, Z = 2.022, *p* = 0.043).

To summarize the results, rehabilitation globally improved divergence latency and amplitude, particularly in the standing condition, and only in one case is there a difference in the sitting condition (hypo group).

### 3.3. Results on Posture

Table 4 shows three of the parameters of posture measured for which significant effects were observed: mean acceleration of medio-lateral body sway for each of the three testing conditions. For the active vergence condition, the normalized area NA and mean power frequency (MPF) together withthe mean latency and amplitude in the standing position are shown (see Table 2); vergence and posture were not recorded simultaneously.

**Figure 7:**
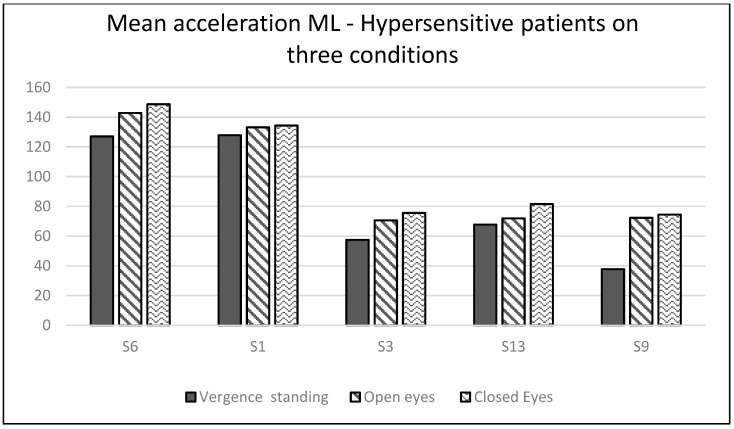
Medio-lateral accelerations during the three testing conditions: vergence in standing position, fixation in standing position with eyes open, and eyes closed conditions in organic patients.

The bold values indicate the results of the patients with vertigo-related dysfunctions of hyper. Considering all patients, they did not show any significant results. Considering the two groups separately reveals some interesting observations. For the hyper group (see Figure 7), one observes that the medio-lateral body sway was lower when actively performing vergence eye movements than when fixating with eyes open or closed.

**Figure 8:**
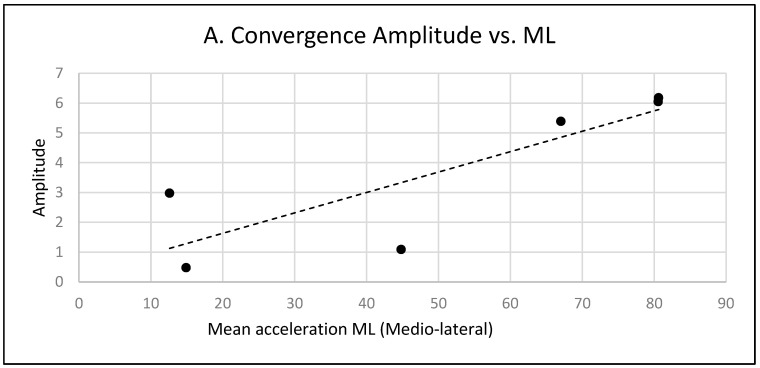

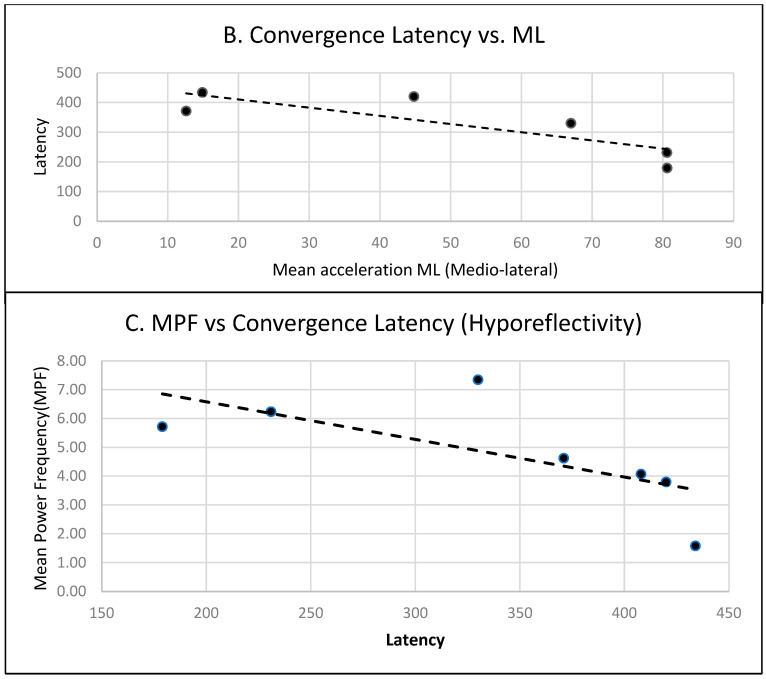
(**A**): Correlation between medio-lateral acceleration and amplitude of convergence on hyporeflectivity patients. (**B**): Correlation between medio-lateral acceleration and latency of convergence on hyporeflectivity patients. (**C**): Correlation between mean power frequency and latency of convergence on hyporeflectivity patients.

For the hypo group with an organic origin of vertigo, postural parameters did not show differences between testing conditions, but there was a significant correlation between posture parameters and latency or amplitude of vergence eye movements. The medio-lateral body sway is higher in patients with larger amplitude of convergence, see Figure 8A, but it is lower in patients with shorter latency of convergence, see Figure 8B. In other words, the larger the amplitude of convergence, the higher the medio-lateral body sway, but the faster the convergence initiation, the less the medio-lateral sway. Additionally, correlation finds that between the latency of convergence and the mean power frequency, the longer the latency of the convergence, the lower the mean power frequency, see Figure 8C.

## 4. Discussion

The purpose of this study was to investigate the prevalence of vergence problems in patients with a history of vertigo from any origin, and assess the potential for improving vergence following rehabilitation with a research-based protocol. To better understand the interaction between vergence, vestibular, and postural functions, vergence was assessed in standing and seated positions, as well as posture being measured during fixation versus vergence eye movements tests. Although being a pilot study, it yielded several interesting results.

Vergence problems exist in 39% of the population (see [31], Kapoula et al., 2016), but in the presence of vestibular pathology, as in this study, nearly 100% of patients have problems with vergence. Eight of the studied patients experienced an improvement in the quality of their vergences: in only four REMOBI neuro-rehabilitation sessions, the latency times decreased, the amplitudes or the speed increased, and the movements became more regular. Patients report better comfort in their relationship to space and their ability to move in crowded spaces such as shopping centers. The benefits were particularly significant in patients with vestibular asthenopia and Meniere’s disease.

These overall quantitative measurements and results showed improvements particularly for divergence, and more particularly in the standing position. This is important as, indeed in vertigo patients, their fear somehow restricts the transitions from near to far space, and this is done with the divergence movement before even undertaking actions.

Thus, the main results show that transition from near to far space is done with better amplitude and shorter latency, i.e., the time of programming the movement. The fact that all these improvements are seen mostly in the standing position is also important because it indicates some part of a multi-sensory integration of vergence vestibular and somatosensory information mediating the improvement. This observation aligns with reports from several subjects mentioning better body self-control and confidence when ambulating in spaces such as in shopping centers.

Further exploration of the postural control in relation to eye movement instructions shows that patients with a functional origin of vertigo present lower acceleration of ML body sway when actively doing vergence eye movements between a near and a far target at their own pace. This is in line with prior findingsin elderly patients (Matheron et al. [33]), or even in patients with bilateral vestibular loss (Kapoula et al. [34]). Thus, patients with a functional origin of vertigo clearly can benefit from active vergence eye movements. Further training of vergence eye movements in such patients could be beneficial for their postural control and equilibrium, which deserves further investigation.

The results from patients with an organic origin suggest rather a perturbed link between eye movements and posture. For instance, the larger the convergence amplitude, the higher the acceleration of ML sway. Yet, the shorter the latency of convergence, the lower the acceleration of ML sway. The mean power frequency is believed to be an indicator of the energy needed to keep posture stable (see [34]). Presumably, the shorter the latency of convergence is, the less energy is needed to keep the body stable. It is unfavorable that we do not know what happened after rehabilitation for the posture. Further studies are needed with larger patient cohorts and recordings of eye movements and posture together before and after rehabilitation.

## 5. Conclusions

Patients who experienced vertigo, regardless of their origin, consistently showed vergence disorders. This mediocre quality of vergence eye movements observed in the course of a vertiginous episode, for some cases many years after the onset of vertigo, is explained by the reciprocity highlighted between vestibular function and that of vergences. A specific rehabilitation protocol of vergence movements (double-step protocol) with the visual acoustic device REMOBI allows one to recover the lost quality of these vergences in terms of temporality, kinetics, and amplitude. This results in improvement of the postural and ambulation comfort in space, as reported by the patients. The vestibular reeducation devoted to the treatment of vertigo should include neuro-training of vergence eye movements to targets displayed naturally in 3D space rendering the transition between near and far spaces smoother. Theoretically, vergence disorders in these patients confirm the natural symbiosis that exists between vergence and vestibular function. Persistent abnormalities of vergence may contribute to the maintenance or recurrence of vertigo symptoms. The neuro-rehabilitation protocol with REMOBI (standing-seated) is integrative, stimulating both the binocular visuo-motor system, the cortical (frontal parietal) and subcortical network controlling this motor function, the musculoskeletal system, and vestibular system as a whole, using stimuli in real space in three dimensions, as in everyday life. It allows the improvement of the temporality of the vergence and thus a better interaction between vestibular and postural functions. Overall, the results confirm both hypotheses, i.e., vergence problems are frequent and dependent on posture (seated standing) in vertigo patients, but they can be improved via appropriate training.

## 6. Patents

REMOBI (Patent US885 1669, WO2011073288), Functional differential exploration and rehabilitation of binocular motor coordination in individuals suffering from vertigo, CNRS, PCT, WO2022/074132, Europe 7 October 2021.

## Figures and Tables

**Table 1:** Patient profile and clinical characteristics as sex and age, major complaints, seniority, fusional amplitudes, NPC (in cm) near point of convergence, and vestibular status. For orthoptic tests, normative values as reported by Kapoula et al. [32] are: NPC < 10 cm, stereo acuity < 60′′, far exophoria 0–2 prism diopters [pD], near exophoria lower than 6 pD, far divergence 5–9 pD, near divergence 15–23 pD, far convergence 15–23 pD, and near convergence 18–24 pD.

Sex and Age	Major Complaints	History	Fusion Amplitudes	NPC (cm)	Vestibular Status
M 76	Positional vertigo	3 weeks	strabismic	8	Hypersensibility
F 38	VestibularAsthenopia	5 weeks	C′25 C18 D′10 D2	5	Hypersensibility
M 79	Vertigo and instability	3 years	C′18 C8 D′12 D4	7	Spontaneous right
M 58	Vertigo and diplopia	2 weeks	C′30 C20 D′12 D4	10	Hyporeflectivity left
F 55	Vestibular Asthenopia	6 weeks	C′35 C8 D′12 D4	8	Hypersensibility
F 63	Discrete vertigo	8 years	C′20 C8 D′12 D4	7	Hyporeflectivity right
F 52	VestibularAsthenopia	2 months	C′35 C18 D′12 D10	5	Nys. Sp. Vert. Inf. idiop.
M 64	Positional vertigo	10 days	C′20 C16 D′10 D2	5	Hypersensibility
M 40	Discrete vertigo	3 weeks	C′14 C8 D′10 D2	5	Hyporeflectivity left
F 53	Discrete vertigo	15 days	C′30 C4 D′6 D2	5	Hypersensibility
F 33	VestibularAsthenopia	3 months	C′18 C18 D′12 D4	3	Hypersensibility
F 48	Discrete vertigo	5 months	C′35 C14 D′8 D2	3	Hyporeflectivity right
M 61	Chronic vertigo	4 years	C′30 C4 D′6 D4	15	Hyporeflectivity left
M 65	Chronic vertigo	8 years	C′0 C10 D′10 D10	7	Hyporeflectivity right

**Table 2:** Parameters of convergence eye movements of before (**A**) and after (**B**) eye movement rehabilitation with REMOBI. The patients which are in bold had functional problems (hypersensibility patients) and the other patients had organic pathology (hyporeflectivity patients).

**(A) CONVERGENCE**							
**SUBJECTS**		**BEFORE**					
**ID**	**Condition**	**Amplitude**	**Latency**	**No of Lat Measures**	**Duration**	**AVelocity**	**PVelocity**
BM	Sit	**0.44 ± 0.03**	**450 ± 82**	15/20	**9 ± 2**	**51 ± 16**	110 ± 26
	Stand	**2.98 ± 0.99**	**371 ± 90**	13/20	**11 ± 3**	**56 ± 15**	**202 ± 57**
**BA**	Sit	**0.83 ± 0.14**	**380 ± 89**	14/20	**49 ± 12**	**12 ± 4**	**34 ± 8**
	Stand	5.11 ± 1.32	258 ± 46	7/20	469 ± 16	**12 ± 1**	54 ± 11
CP	Sit	6.26 ± 0.63	183 ± 24	19/20	285 ± 56	16 ± 4	**36 ± 8**
	Stand	6.18 ± 0.71	179 ± 33	19/20	372 ± 67	**16 ± 2**	**41 ± 7**
CR	Sit	**1.53 ± 0.37**	**388 ± 97**	19/20	**8 ± 3**	15 ± 7	**39 ± 10**
	Stand	**1.09 ± 0.28**	**420 ± 102**	20/20	**39 ± 7**	16 ± 4	**34 ± 9**
**DA**	Sit	5.7 ± 0.92	179 ± 33	6/20	**141 ± 30**	31 ± 7	85 ± 18
	Stand	**0.18 ± 0.03**	**446 ± 108**	10/17	**7 ± 2**	28 ± 10	**26 ± 7**
**DV**	Sit	**4.22 ± 1.18**	**311 ± 60**	17/20	**175 ± 31**	18 ± 5	57 ± 13
	Stand	6.87 ± 0.55	252 ± 38	20/20	373 ± 92	16 ± 3	64 ± 11
FM	Sit	8.75 ± 1.87	228 ± 41	16/20	**13 ± 4**	**8 ± 3**	**44 ± 7**
	Stand	6.05 ± 0.96	231 ± 33	16/20	244 ± 64	**16 ± 3**	**43 ± 8**
FOM	Sit	**4.62 ± 0.92**	**395 ± 66**	14/20	227 ± 77	20 ± 5	67 ± 9
	Stand	5.39 ± 1.22	330 ± 94	17/20	**132 ± 26**	**4 ± 1**	**21 ± 5**
**GJ**	Sit	6.7 ± 1.86	284 ± 56	15/20	**104 ± 44**	36 ± 6	128 ± 24
	Stand	8.37 ± 1.89	233 ± 42	11/20	290 ± 52	26 ± 8	72 ± 21
**MJ**	Sit	**0.27 ± 0.08**	**417 ± 114**	13/20	**58 ± 22**	**2 ± 1**	**4 ± 1**
	Stand	**0.2 ± 0.08**	**408 ± 57**	13/16	**38 ± 9**	**6 ± 2**	**19 ± 3**
JL	Sit	6.14 ± 1.05	235 ± 49	17/20	**146 ± 30**	29 ± 7	70 ± 17
	Stand	8.36 ± 1.38	252 ± 47	18/20	**164 ± 30**	27 ± 6	92 ± 18
**PF**	Sit	**2.68 ± 0.41**	264 ± 38	20/20	**60 ± 10**	29 ± 9	95 ± 28
	Stand	**0.59 ± 0.15**	**378 ± 57**	10/20	**112 ± 59**	**5 ± 4**	**12 ± 6**
WB	Sit	**0.14 ± 0.06**	**436 ± 90**	19/20	**8 ± 2**	**7 ± 2**	**9 ± 3**
	Stand	**0.48 ± 0.09**	**434 ± 82**	18/20	**51 ± 6**	**10 ± 4**	**36 ± 13**
Mean	Sit	3.713	319.23		98.692	21.076	59.846
	Stand	3.98	322.46		177.07	18.30	55.07
Normal Values		7.76 ± 1.36	194 ± 48		343 ± 87	26 ± 6	89 ± 27
**(B) CONVERGENCE**							
**SUBJECTS**		**AFTER**					
**ID**	**Condition**	**Amplitude**	**Latency**	**No of Lat Measures**	**Duration**	**AVelocity**	**PVelocity**
BM	Sit	**0.23 ± 0.1**	**487 ± 67**	9/20	**48 ± 7**	**6 ± 2**	**11 ± 4**
	Stand	**0.28 ± 0.07**	**466 ± 95**	13/20	**7 ± 2**	**7 ± 3**	**10 ± 3**
**BA**	Sit	6.59 ± 0.69	**309 ± 56**	18/20	432 ± 48	**16 ± 3**	59 ± 15
	Stand	6.3 ± 1.5	228 ± 27	13/20	203 ± 59	19 ± 4	70 ± 19
CP	Sit	**3.3 ± 0.74**	208 ± 24	19/20	276 ± 58	**11 ± 2**	**22 ± 4**
	Stand	5.32 ± 1.04	214 ± 20	10/20	218 ± 59	18 ± 5	59 ± 15
CR	Sit	**3.98 ± 0.32**	254 ± 32	15/20	206 ± 63	**13 ± 3**	**37 ± 8**
	Stand	6.12 ± 0.48	245 ± 25	14/20	**174 ± 37**	26 ± 6	84 ± 16
**DA**	Sit	**3.5** ± 0.13	263 ± 49	18/20	**60 ± 11**	22 ± 6	64 ± 19
	Stand	**4** ± 0.31	263 ± 47	18/20	**59 ± 6**	23 ± 4	59 ± 12
**DV**	Sit	**3.84 ± 0.59**	298 ± 48	17/20	**157 ± 50**	17 ± 4	65 ± 11
	Stand	6.73 ± 0.54	**300 ± 43**	19/20	286 ± 69	19 ± 3	78 ± 12
FM	Sit	**4.55 ± 0.9**	254 ± 36	14/20	351 ± 60	**12 ± 1**	**45 ± 9**
	Stand	**4.42 ± 0.64**	278 ± 41	17/20	**173 ± 44**	19 ± 5	64 ± 12
FOM	Sit						
	Stand						
**GJ**	Sit	6.76 ± 0.93	275 ± 45	16/20	203 ± 36	31 ± 7	88 ± 16
	Stand	8.17 ± 1.3	263 ± 40	19/20	**151 ± 28**	49 ± 10	150 ± 27
**MJ**	Sit	**0.21 ± 0.07**	**455 ± 112**	14/20	**9 ± 1**	**4 ± 1**	**15 ± 5**
	Stand	**0.4 ± 0.21**	**412 ± 107**	15/20	**58 ± 14**	**8 ± 2**	**9 ± 3**
JL	Sit	8.76 ± 1.13	252 ± 42	19/20	470 ± 42	17 ± 4	58 ± 11
	Stand	7.44 ± 0.8	261 ± 37	19/20	**142 ± 13**	32 ± 8	87 ± 13
**PF**	Sit	**4.21 ± 1.29**	**308 ± 45**	15/20	251 ± 60	**10 ± 2**	**30 ± 7**
	Stand	6.83 ± 1.24	274 ± 22	20/20	294 ± 74	19 ± 2	86 ± 13
WB	Sit	**0.36 ± 0.08**	**368 ± 105**	9/20	**9 ± 1**	30 ± 5	86 ± 23
	Stand						
Mean	Sit	3.347	305		190.84	14.61	44.76
	Stand	4.505	305.16		151.583	20.66	65.91
Normal Values		7.76 ± 1.36	194 ± 48		343 ± 87	26 ± 6	89 ± 27

**Table 3:** Parameters of divergence eye movements of before (**A**) and after eye movement rehabilitation with REMOBI (**B**). The patients which are in bold had functional problems (hypersensibility patients) and the other patients had organic pathology (hyporeflectivity patients).

**(A) DIVERGENCE**							
**SUBJECTS**		**BEFORE**					
**ID**	**Condition**	**Amplitude**	**Latency**	**No of Lat Measures**	**Duration**	**AVelocity**	**PVelocity**
BM	Sit	3.19 ± 1.05	**375 ± 82**	12/20	**42 ± 6**	**54 ± 8**	**124 ± 38**
	Stand	5.31 ± 1.61	**429 ± 83**	11/20	**18 ± 4**	**84 ± 19**	**178 ± 50**
**BA**	Sit	4.4 ± 0.53	207 ± 27	15/20	346 ± 59	12 ± 3	32 ± 7
	Stand	**3.46 ± 0.4**	207 ± 47	7/20	285 ± 47	**11 ± 3**	33 ± 3
CP	Sit	4.48 ± 0.75	214 ± 36	17/20	415 ± 108	**10 ± 2**	**30 ± 3**
	Stand	4.91 ± 0.38	250 ± 41	20/20	390 ± 88	**11 ± 2**	36 ± 5
CR	Sit	**0.91 ± 0.24**	**416 ± 111**	18/20	**46 ± 6**	26 ± 9	55 ± 13
	Stand	**0.69 ± 0.22**	**483 ± 149**	16/20	**44 ± 5**	14 ± 4	43 ± 12
**DA**	Sit	4.35 ± 0.45	187 ± 31	15/20	274 ± 30	15 ± 2	38 ± 8
	Stand	**0.06 ± 0.01**	**387 ± 88**	10/18	**10 ± 2**	**2 ± 0**	**25 ± 9**
**DV**	Sit	**4.14 ± 0.61**	206 ± 26	14/20	274 ± 56	15 ± 3	40 ± 8
	Stand	4.84 ± 0.46	182 ± 26	20/20	287 ± 30	16 ± 2	40 ± 5
FM	Sit	4.87 ± 1.1	207 ± 34	13/20	402 ± 78	**10 ± 2**	**30 ± 4**
	Stand	**3.7 ± 0.66**	282 ± 57	18/20	305 ± 68	**11 ± 2**	**31 ± 4**
FOM	Sit	5.11 ± 0.35	273 ± 72	17/20	390 ± 67	**12 ± 2**	32 ± 7
	Stand	4.69 ± 0.44	226 ± 39	18/20	362 ± 53	13 ± 2	**30 ± 4**
**GJ**	Sit	**4.25 ± 1.31**	**387 ± 106**	11/20	**7 ± 2**	**39 ± 9**	**121 ± 28**
	Stand	**4.08 ± 0.75**	**398 ± 75**	12/20	299 ± 68	12 ± 3	37 ± 9
**MJ**	Sit	**3.85 ± 0.6**	**449 ± 84**	14/20	**50 ± 63**	**3 ± 1**	**22 ± 5**
	Stand	**0.28 ± 0.09**	**582 ± 136**	13/19	**67 ± 21**	**4 ± 1**	**8 ± 2**
JL	Sit	5.14 ± 1.08	**301 ± 53**	18/20	**8 ± 2**	**515 ± 161**	**1437 ± 309**
	Stand	5.39 ± 0.81	**285 ± 38**	20/20	**32 ± 8**	**395 ± 113**	**1763 ± 440**
**PF**	Sit	**2.16 ± 0.6**	**322 ± 82**	17/20	**51 ± 5**	**39 ± 12**	**109 ± 35**
	Stand	**1.51 ± 0.38**	**349 ± 85**	11/20	**47 ± 3**	**31 ± 8**	**90 ± 26**
WB	Sit	**1.07 ± 0.32**	**434 ± 83**	14/20	**130 ± 40**	12 ± 3	31 ± 8
	Stand	**1.96 ± 0.46**	**410 ± 121**	17/20	220 ± 72	14 ± 3	36 ± 7
Mean	Sit	3.68	306		187.30	58.61	161.61
	Stand	3.14	343.84		182	47.53	180.76
Normal Values		5.32 ± 0.42	203 ±27		306 ± 33	16 ± 1	41 ± 5
**(B) DIVERGENCE**							
**SUBJECTS**		**AFTER**					
**ID**	**Condition**	**Amplitude**	**Latency**	**No of Lat Measures**	**Duration**	**AVelocity**	**PVelocity**
BM	Sit	**0.86 ± 0.36**	309 ± 90	11/39	**8 ± 3**	15 ± 3	**14 ± 3**
	Stand	**2.5 ± 0.57**	331 ± 82	14/39	**163 ± 40**	15 ± 3	42 ± 9
**BA**	Sit	4.64 ± 0.26	166 ± 29	13/39	331 ± 33	13 ± 2	33 ± 4
	Stand	4.45 ± 0.47	171 ± 31	17/39	280 ± 51	15 ± 3	38 ± 7
CP	Sit	5.23 ± 0.35	191 ± 24	18/20	**504 ± 104**	**3 ± 0**	**26 ± 5**
	Stand	5.57 ± 0.37	224 ± 35	18/20	424 ± 96	**12 ± 2**	40 ± 5
CR	Sit	**2.74 ± 0.26**	210 ± 27	20/20	**216 ± 40**	**11 ± 2**	41 ± 6
	Stand	**3.8 ± 0.4**	229 ± 42	19/20	247 ± 43	14 ± 2	50 ± 7
**DA**	Sit	**5 ± 0.72**	265 ± 66	17/20	**140 ± 27**	**11 ± 2**	36 ± 10
	Stand	**4 ± 0.49**	260 ± 58	16/20	**180 ± 45**	14 ± 3	38 ± 8
**DV**	Sit	**4.12 ± 0.45**	185 ± 40	19/39	257 ± 41	15 ± 3	40 ± 7
	Stand	5.27 ± 0.34	186 ± 27	19/39	320 ± 45	15 ± 2	38 ± 4
FM	Sit	**3.56 ± 0.57**	289 ± 69	20/20	252 ± 64	12 ± 3	38 ± 5
	Stand	**3.69 ± 0.69**	274 ± 52	20/20	249 ± 41	14 ± 2	42 ± 6
FOM	Sit	4.92 ± 0.15	264 ± 61	19/20	328 ± 45	15 ± 2	38 ± 4
	Stand	5.54 ± 0.42	208 ± 35	18/20	395 ± 55	13 ± 2	34 ± 4
**GJ**	Sit	4.89 ± 0.96	**341 ± 87**	15/20	297 ± 63	15 ± 3	49 ± 6
	Stand	5.63 ± 0.78	**323 ± 66**	17/20	**225 ± 43**	20 ± 4	**67 ± 11**
**MJ**	Sit						
	Stand	**1.08 ± 0.33**	273 ± 80	14/20	**146 ± 22**	**3 ± 1**	**16 ± 4**
JL	Sit	**6.32 ± 0.8**	219 ± 50	17/40	437 ± 71	14 ± 1	39 ± 5
	Stand	5.87 ± 0.75	210 ± 37	14/39	325 ± 63	17 ± 2	42 ± 4
**PF**	Sit	**3.3 ± 0.5**	**308 ± 46**	17/20	254 ± 51	**12 ± 2**	35 ± 5
	Stand	4.64 ± 0.73	257 ± 45	18/20	244 ± 39	16 ± 3	51 ± 10
WB	Sit	**0.56 ± 0.17**	**366 ± 107**	18/39	**9 ± 3**	**42 ± 13**	**85 ± 24**
	Stand						
Mean	Sit	3.38	261		234.07	14.69	38.84
	Stand	4.33	245.5		266.5	14	41.5
Normal Values		5.32 ± 0.42	203 ±27		306 ± 33	16 ± 1	41 ± 5

**Table 4:** Values of medio-lateral acceleration (ML), normalized area (NA), and mean power frequency (MPF) along with latency and amplitude of convergence eye movements, postural data from standing convergence, then latency and amplitude of standing convergence.

Patients	Acceleration Mean ML			Posture Parameters During Vergence Test		Convergence Eye Movement Parameters	
	Vergence Standing	Open Eyes	Closed Eyes	NA	MPF	Latency	Amplitude
**DV**	127.812928	133.14236	134.286	5.60	6.77	252	6.87
FM	80.5680144	55.800537	56.0353	9.78	1.58	231	6.05
**GJ**	57.4096559	70.483845	75.5005	18.36	4.65	233	8.37
MJ		129.11914	121.557	58.23	4.07	408	0.2
WB	14.9056372	27.14229	32.1247	0.14	3.79	434	0.48
**BA**	127.019082	142.76588	148.62	0.55	10.29	258	5.11
CP	80.6089137	74.411761	66.2667	0.88	6.23	179	6.18
CR	44.8039054	5.6714822	7.89027	0.16	5.71	420	1.09
**DA**	37.7262351	72.203417	74.4235	0.44	7.21	446	0.18
FM	67.015078	94.584986	87.5855	0.49	7.34	330	5.39
MB	12.6030973	24.679846	19.437	6.06	4.62	371	2.98
**PF**	67.668139	71.842767	81.5111	0.23	6.68	378	0.59

## Data Availability

The original contributions presented in the study are included in the article, further inquiries can be directed to the corresponding author.
